# One million scans later: the evolution of the I16 material and magnetism beamline at Diamond Light Source

**DOI:** 10.1107/S1600577526006557

**Published:** 2026-08-07

**Authors:** Aly H. Abdeldaim, Rebecca Scatena, James Hawkins, Gareth Nisbet, Daniel G. Porter, Graeme Barlow, Andy Marshall, Brian Nutter, Guillaume Beutier, Stephen P. Collins, Alessandro Bombardi

**Affiliations:** a Diamond Light Source Harwell Science and Innovation Campus Didcot Oxfordshire United Kingdom; b CERN Geneva Switzerland; c University Grenoble Alpes CNRS, Grenoble INP, SIMaP Grenoble France; European XFEL, Germany

**Keywords:** X-ray beamline, REXS, NRXS, BCDI, GI-WAXS

## Abstract

Here, we review the capabilities of beamline I16 at Diamond Light Source after nearly 20 years of operation and continuous development. I16 is an X-ray scattering beamline operating in the 2.46–16 keV energy range with energy scans with a resolution of 1 meV. The beamline is equipped with a six-circle kappa diffractometer, offering flexible experimental geometries and support for a variety of ancillary equipment. Available equipment includes cryostats and cryofurnaces that span temperatures from 4.2 K to 700 K, which can be combined with applied electric fields, permanent magnets up to 1 T, and uniaxial strain cells. A range of detectors is mounted on the diffractometer arm, including a photon-counting area detector and an avalanche photodiode, both compatible with crystal analyzers for polarization analysis. Diamond single-crystal phase retarders provide full control of the incident X-ray polarization. These capabilities establish I16 as a state-of-the-art instrument for resonant and non-resonant X-ray scattering, multi Bragg coherent diffraction imaging, and grazing-incidence small- and wide-angle X-ray scattering. Together, they allow for studies of complex electronic, magnetic and structural phenomena in single crystals and thin films. We also outline the software infrastructure supporting experiment planning, data acquisition and rapid on-the-fly data analysis, and discuss ongoing developments and the anticipated benefits of the upgrade to a fourth-generation source.

## Abstract 2

Beamline I16 at Diamond Light Source is a versatile X-ray scattering instrument that combines tunable X-ray energy and polarization, flexible diffraction geometries, and advanced sample environments for studies of electronic, magnetic, and structural phenomena.

## 1. Introduction

Development in experimental physics is inseparable from the evolution of its experimental tools. Scattering techniques have been a cornerstone of this progress, as advances in theory and the continual discovery of newly synthesized materials demand increasingly sophisticated access to subtle complex collective and magnetic phenomena, often in small samples and under extreme conditions. The I16 beamline at Diamond Light Source is ideally suited for such studies of functional and quantum materials, offering a suite of resonant (REXS) and non-resonant (NRXS) elastic X-ray scattering techniques in addition to coherent diffraction tools, amongst others. Together, these can give simultaneous access to charge, spin, orbital and multipolar physics along with structural diffraction imaging capabilities across the tender to hard X-ray regime. Following the recent milestone of the one millionth scan on I16, we review the beamline’s current status beyond the initial conference proceeding (Collins *et al.*, 2010 ▸).

The I16 beamline was one of the first to welcome users at Diamond Light Source and has operated continuously since 2007. The beamline has been in constant evolution since then, resulting in a setup that is optimized to make full use of the third-generation light source beam supporting all requirements for state-of-the-art REXS and NRXS experiments. Its capabilities include a high-photon beam flux at sample with tunable energy and incident beam polarization, low harmonic contamination, full control of the scattering geometry, and an in-house-built polarization analyser stage for the diffracted beam. Combined with a suite of low-noise area detectors and sample environments offering controlled temperature, magnetic and electric fields, and applied uniaxial strain, these features make I16 well suited for investigating complex ordering phenomena and exotic scattering processes in micrometre-sized crystals, multilayers, and ultrathin films across the *K*-, *L*-, and *L*- and *M*-absorption edges of 3*d*, 4*d* and 5*d* transition metals, respectively, as well as actinides and lanthanides.

Beyond this, I16 supports a broad range of additional specialized experimental techniques. The length of the beamline, low beam divergence and near-windowless beam path retain a highly coherent beam in the medium to tender X-ray energy range. This allows for various coherent diffraction experiments including Bragg coherent diffraction imaging (BCDI) and X-ray photon correlation spectroscopy (XPCS), that can be combined with low vibration sample environments. Additionally, the open geometry of the kappa diffractometer and large area detectors further support other techniques such as reflectivity and wide- and small-angle X-ray scattering (WAXS, SAXS). The beamline also serves as a platform for developing new methodologies, including studies of diffuse multiple scattering (Nisbet *et al.*, 2015 ▸), parametric down conversion (Sofer *et al.*, 2019 ▸; Sofer *et al.*, 2021 ▸), and others.

The following review is an overview of the scientific and instrumental scope of I16 and describes its technical capabilities, experimental configurations and available instrumentation.

## 2. Beamline overview/optical configuration

**Figure 1:**
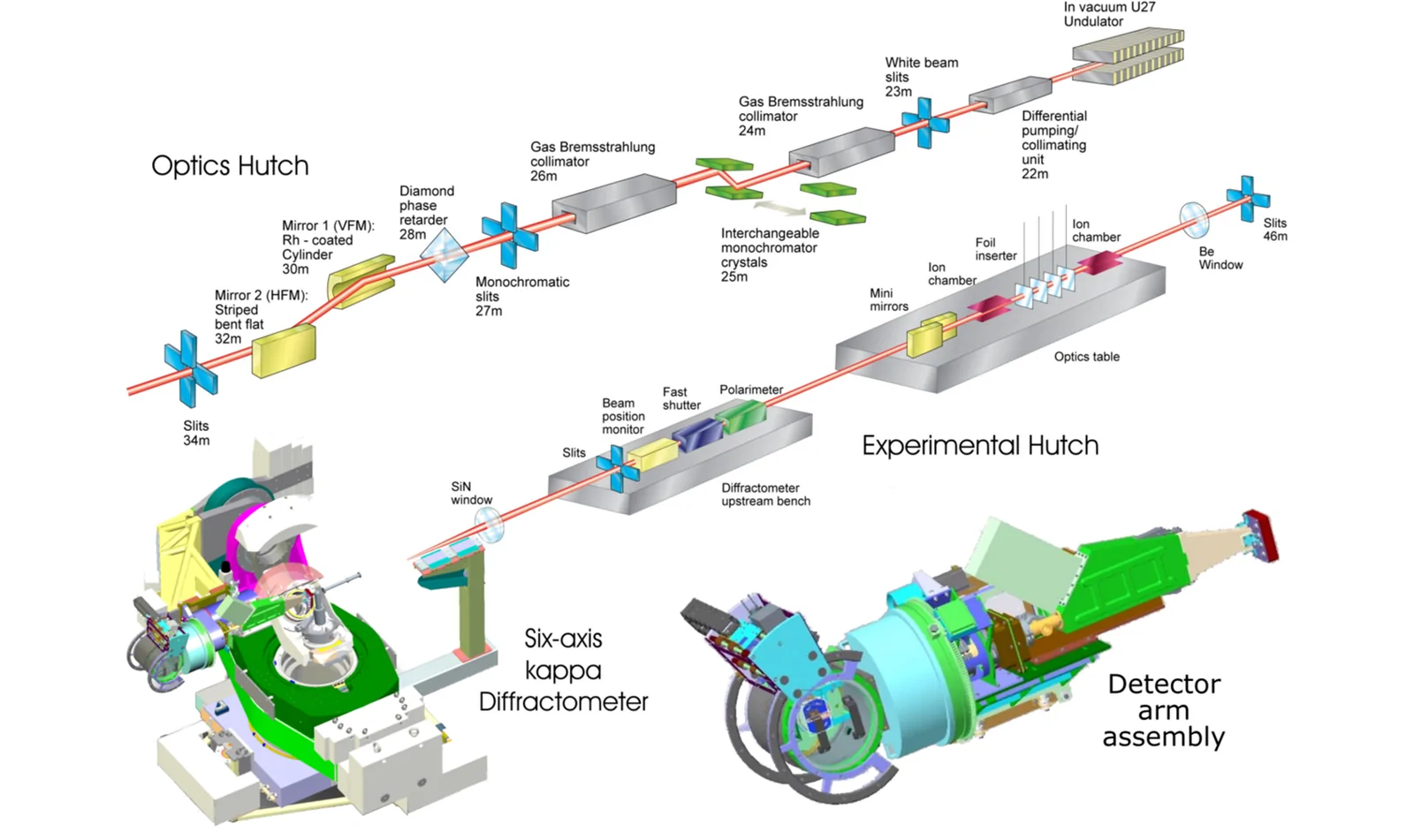
Layouts of the optics and experimental hutches of I16 along with the detector arm assembly.

I16 delivers a high brightness and highly coherent beam with full control over the incident polarization, high resolution, polarization analysis, and low harmonic contamination. These performance specifications are achieved through careful consideration of the beamline length, positioning, type and the quality of the optical components in the long windowless in-vacuum section extending to the source (Fig. 1 ▸). Approximately three meters before the sample, a 150 µm-thick Be window separates the high-vacuum section from a 1 bar He section. The He section is maintained to allow for the operation of ionization detectors and is followed by a 1 µm-thick Si_3_N_4_ beam exit window. Diagnostics with diodes and fluorescence screens, along with attenuator foils, slits and calibration foils can be remotely inserted downstream of most active optical elements for the beam monitoring, alignment, calibration and and intensity control.

### 2.1. Source

The X-ray source for beamline I16 is an in-vacuum Diamond Light Source U27b undulator insertion device supplied by Danfysik and discussed by Patel *et al.* (2017 ▸). The undulator is 2 m long and uses Sm_2_Co_17_ permanent magnets, with a minimum gap of 5 mm, peak field of 0.98 T and a period length of 27 mm. By selecting odd harmonics, the insertion device produces an intense, tunable X-ray beam with linear horizontal polarization that delivers up to 10^15^ photons s^−1^ (0.1% bandwidth)^−1^ across a continuous energy range of 2.46 keV to 16 keV.

### 2.2. Monochromator

The precise photon energy is selected using a cryogenically indirectly cooled Si(111) channel-cut crystal with a 7 mm gap (Khosroabadi *et al.*, 2022 ▸). For energies above 3.1 keV, the monochromator operates in a two-bounce mode, while below 3.1 keV, the larger 2θ angle requires four bounces of the photon beam. To compensate for the resulting vertical beam shift during energy changes, all active optical elements downstream of the monochromator are automatically vertically adjusted when energy changes are larger than 20 eV or based on a desired threshold. The channel cut delivers a monochromatic beam with high brightness and excellent stability across the full energy range supported by the undulator. It allows for rapid energy tuning and scans with an energy resolution of Δ*E*/*E* ≃ 10^−4^. A new monochromator system is currently being designed for Diamond-II and is further discussed in Section 6.

### 2.3. X-ray phase retarders

**Figure 2:**
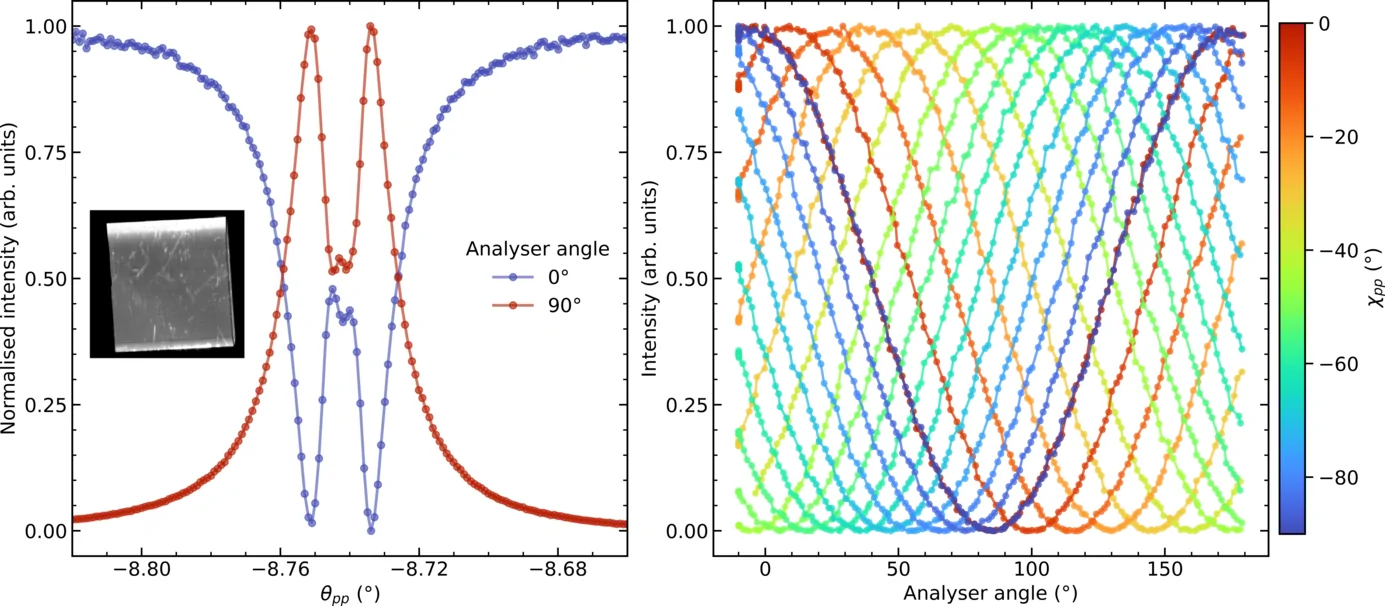
Left: intensity as a function of phase-plate Bragg angle (θ_pp_) for a 400 µm-thick diamond phase plate at 6.971 keV, measured at the (−1, 1, −1) reflection. The polarization of the transmitted intensity was analysed using a Cu (2, 2, 0) polarization analyser at analyser angles of 0° (blue) and 90° (red). Left inset: monochromatic topographical image of the 1000 µm-thick diamond phase plate highlighting the excellent quality of the monosectorial plate. Right: continuous rotation of linearly polarized light with a 1000 µm-thick diamond phase plate at 7 keV as a function of the rotation angle of the phase retarder crystal, χ_pp_.

After the monochromator, in-vacuum X-ray phase retarders can be remotely inserted into the beam to fully control the incident polarization (Fig. 2 ▸). This includes linear polarization at arbitrary angles relative to the electron orbit, as well as left- and right-handed circular polarization over a broad energy range of 3.1 keV to 13 keV. The use of single crystals in transmission to modify the X-ray polarization has been described by several authors (*e.g.* Scagnoli *et al.*, 2009 ▸; Giles *et al.*, 1995 ▸) and falls beyond the scope of this review. However, we detail how this was implemented on beamline I16. The single-crystal phase retarders used on I16 are high-quality type IIa diamond plates of various thicknesses, used for different incident energies and an Si membrane disk for use below 3.1 keV. These crystals are mounted on two low-stress holders, each providing independent vertical translation for selecting the desired plate or removing it from the beam, as well as independent rotation to control the incidence angle with respect to the beam. The holders share a common centre of rotation about the beam axis and are installed on a Huber stage that provides mechanical stability. The use of encoders further ensures precise alignment and reproducibility. The Huber stage is installed on a motorized table with motors allowing for translation, height, yaw and pitch control. The first holder accommodates five diamond plates with thicknesses of 1500, 1000, 400, 200 and 100 µm, while the second contains diamond plates of 2000, 200 and 100 µm thickness, together with a 10 µm Si membrane. All plates were pre-oriented and characterized by X-ray topography on beamline B16 at Diamond Light Source before installation. The diamond crystals share a similar orientation and shape, with long edges aligned along the 〈1,1,0〉 direction and flat faces normal to 〈0,0,1〉. In this geometry, both {1,1,1} and {2,2,0} reflections can be accessed in either symmetric or asymmetric Laue geometry. The thin Si membrane has its surface normal aligned with the 〈1,1,1〉 direction. Plate selection depends on the incident beam energy, which influences both the transmission through the phase retarders and the angular separation between their working points, determined by the plates’ effective thickness and angle of incidence. In general, a significant offset from the quarter-wave condition (>0.005°) and a transmitted beam intensity above 10% are desirable to achieve high polarization rates. The choice of reflection is also based on the minimization of multiple scattering. Polarization efficiency can be increased by using two plates sequentially on the incoming beam that is made possible by the dual holder configuration described above. Nonetheless, a single phase plate is often sufficient to achieve high polarization rates and is easier to align. The optical path symmetrization discussed by Scagnoli *et al.* (2009 ▸) and Paolasini *et al.* (2007 ▸) is not typically a decisive factor.

### 2.4. Focusing mirrors

The beam focusing system consists of two horizontally deflecting mirrors. This completely horizontally deflecting configuration was implemented to decouple the aberration introduced by the mirrors and to preserve the vertical features of the beam. The first mirror is a sagittal cylindrical mirror located 20.5 m from the sample position, with a radius of 96 mm, which provides fixed vertical focusing. The second is a circular mechanical bender positioned 19 m from the sample, used at a nominal tangential radius of 5.79 km. The vertical focusing mirror, positioned at an angle of 4 mrad, has an active area of 1200 mm × 20 mm and is made of single-crystal Si coated with Rh. This mirror has a cumulative sagittal slope error of <20 µrad. The horizontal focusing mirror, positioned downstream and parallel to the first, is bent into a vertical cylindrical shape via a mechanical bender. This mirror has an active area of 1200 mm × 30 mm and is made of an Si single crystal, with a 15 mm-wide Rh-coated stripe. Vertical translation allows for selecting the Si or Rh stripes to optimize reflectivity and harmonic rejection depending on the operating energy. Specifically, the Rh or Si stripes are selected for harmonic rejection above 16 keV and 8 keV, respectively. The tangential radius can be adjusted from 40 km to 3.5 km to control the horizontal beam size. Both mirrors have a surface roughness of <3 Å r.m.s. The vertical focus can also be changed by adjusting the angle of both mirrors. The beamline’s long focusing distance, combined with the two high-quality horizontally deflecting mirrors, achieves a focused beam size of 185 µm (H) × 15 µm (V) at the sample position, which matches the theoretically calculated focus. Alternative beam sizes can be achieved by adjusting the angle of the mirrors and varying the bending radius of the second mirror. Furthermore, the long focusing distance provides a gentle focus, with a high coherent flux with controlled wavefront due to the specific optical design.

### 2.5. Harmonic rejection mirrors

As outlined in Section 2.4, harmonics generated by the monochromator are initially suppressed using one of the main focusing mirrors by selecting either the Rh stripe or uncoated Si. For near-complete harmonic rejection below 8 keV, a pair of horizontally deflecting flat mirrors that are located 1.75 m from the sample can be remotely introduced into the beam path and operate in total external reflection. These mirrors are housed in a 1 bar He atmosphere and feature an active area of 260 mm × 15 mm of single-crystal Si coated with a 50 µm-thick layer of B_4_C with a surface roughness <3 Å r.m.s. The horizontal deflection mirrors are motorized with absolute encoders and can be rotated up to 0.4° and allow for near 100% reflectivity at the desired energy and suppression below 10^−6^ at the third harmonic energies of the monochromator. Suppression of the even harmonics is even larger given that they are forbidden reflections of the Si monochromator.

## 3. Endstation

The principal instrument in the experimental hutch is a six-circle Kappa diffractometer (Newport), capable of precise sample alignment in any orientation relative to the incident beam (Fig. 1 ▸). The diffractometer is operated in Eulerian geometry through *Diffcalc* (Diamond Light Source, 2024*a* ▸) with four sample rotations (η, χ, ϕ, μ) and two detector axes (δ, γ) with static resolutions of 0.001° (Table 1 ▸). *Diffcalc* also allows for the diffractometer to be driven directly in reciprocal space. The open geometry of the Kappa diffractometer offers several advantages over a conventional Eulerian instrument; it allows unobstructed access to large scattering angles in both vertical and horizontal geometries as well as intermediate configurations. The sample stage can accommodate loads of up to 15 kg and is equipped with three (*x*, *y*, *z*) translation stages. The diffractometer is mounted on a granite block equipped with *yz*-translation stages which allow for horizontal and vertical translations in addition to pitch adjustments. These, combined with the sample translations, allow for the alignment of the centre of rotation with respect to the beam position. A sphere of confusion of approximately 70 µm is typically observed, and the beam is delivered on the sample with a stability of approximately 10% of the beam dimensions over week-long experiments.

The detector arm assembly was developed at Diamond Light Source and delivers a static resolution of 0.001° in full-balance mode while supporting loads up to 70 kg (Burt *et al.*, 2018 ▸). Housed fully under vacuum, it starts with a Kapton-windowed nose cone of interchangeable length and houses four permanently mounted X-ray detector systems, each of which can be remotely selected. The first is a Dectris Pilatus3 100 K photon-counting area detector that is directly within the vacuum of the detector arm. It is mounted with an angular offset of 8.8° to the beam path and its surface is inclined by 35° to increase its resolution. This detector has a pixel size of 172 µm (width) × 172 µm (height) with an array size of 487 pixels (width) × 195 pixels (height) and is operable across the full incident photon energy range of I16.

**Figure 3:**
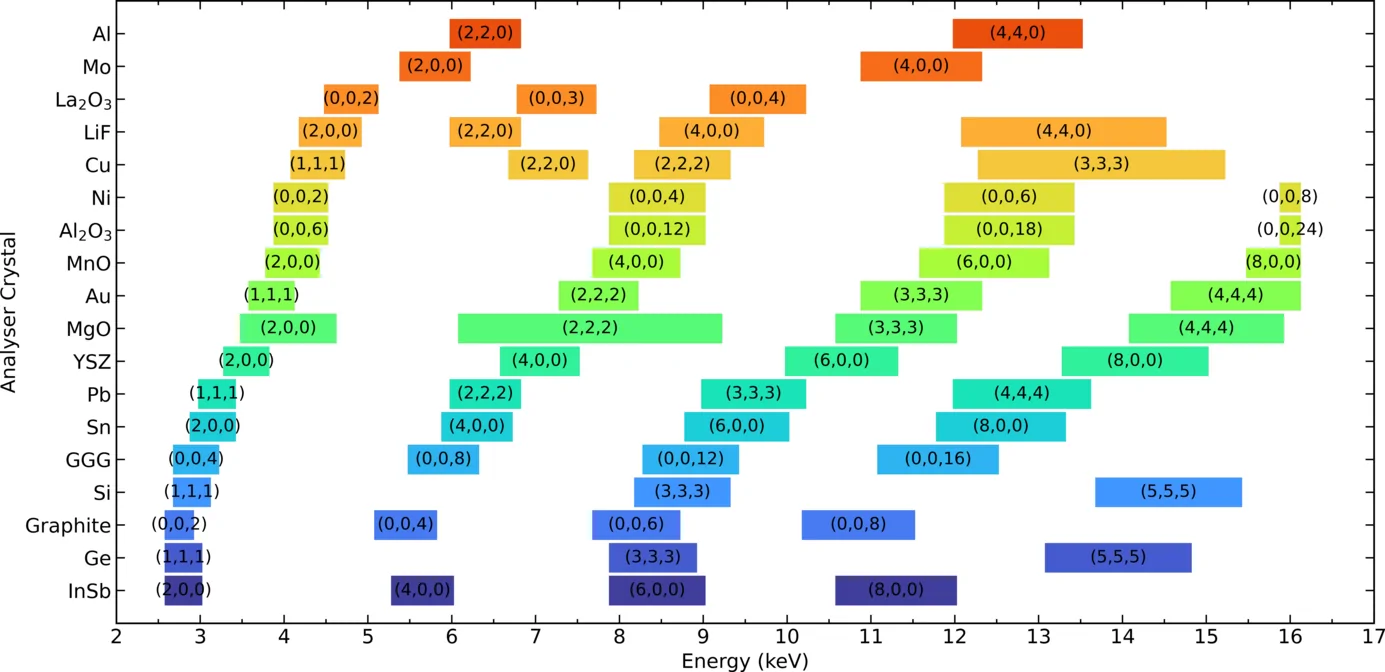
Available single crystals for polarization analysis on I16.

The Pilatus3 100 K is followed by detector slits that define the entrance to the polarization analyser assembly, which hosts an in-vacuum chamber capable of fully rotating about the diffracted beam axis by 360° and incorporates a two-axis diffractometer. Inside the chamber, a high-resolution Medipix QuadMerlin photon-counting area detector is mounted on a 2θ arm. This detector has a pixel size of 55 µm × 55 µm and an array size of 515 pixels × 515 pixels. The θ stage can accommodate analyser crystals or a high-resolution single- or triple-bounce Si〈111〉 analyser that can be easily installed or removed and can be continuously rotated. For polarization analysis, the analyser crystal is typically mounted with a Bragg angle at ∼45° towards the scattered beam, and the detector is positioned at a 2θ angle near 90°. A wide selection of analyser crystals is readily available to match the beamline’s operational energies and to accommodate for samples with different mosaicities, as shown in Fig. 3 ▸. Beyond the vacuum chamber, a secondary 2θ arm and translation bracket house an avalanche photodiode and a current counting Hamamatsu photodiode, located behind a beryllium window in air. These point detectors can be selected for use directly in the diffracted beam or in combination with the polarization analyser. Anti-scatter slits can be optionally mounted in front of the nose cone to minimize diffuse scattering from air and beryllium.

In addition to the permanently mounted detectors, a large Dectris Pilatus 2M photon-counting area detector [pixel size of 172 µm (width) × 172 µm (height) with an array size of 1475 pixels (width) × 1679 pixels (height)] can be installed for a broad range of experiments. Such experiments include grazing incidence small- and wide-angle X-ray scattering but also conventional single-crystal and powder diffraction. This detector can be mounted together with a He-filled flight tube, and is mounted independently of the main detector arm on an extendable table. The sample-to-detector distance can be varied between 500 mm and 2000 mm (or 250 mm to 2000 mm without a flight tube), while the table itself can be manually rotated about the γ axis of the diffractometer.

### 3.1. Ancillary equipment

The large, open geometry of the Kappa diffractometer can accommodate various sample stages and ancillary equipment that allow for temperature control, applied magnetic fields, uniaxial strain and electric fields. Temperature control between 6.5 K and 350 K is routinely achieved using an ARS DE-202SK closed-cycle cryostat, and between 15 K and 700 K using an ARS DE-202AK closed-cycle cryostat. For applications requiring ultra-low vibration, a ColdEdge Stinger closed-cycle cryostat is available and can be operated between temperatures ranging from 4.2 K to 350 K. All cryostats limit the sample ϕ rotation but retain at least 200° of accessible range. All cryostat sample stages operate with one or two Be windows of 0.25 mm thickness each, respectively. An Oxford Diffraction Cryostream cooler is also available and can operate within a temperature range of 90 K to 500 K.

Additional ancillary equipment can be mounted on the ARS DE-202SK cryostat stage, including a CS100 Razorbill strain cell for automated *in situ* uniaxial strain measurements down to approximately 7.5 K, and MC051 Razorbill mechanical strain cells with 800 µm and 500 µm gaps. For experiments requiring magnetic fields, compact permanent magnets capable of generating fields up to 1 T with large reciprocal-space access have been designed in-house. Furthermore, static and oscillating electric fields up to 5 kV with arbitrary waveforms can be applied at room temperature. The list of ancillary equipment constantly evolves, and users are encouraged to visit the beamline website for the latest updates (Diamond Light Source, 2026*a* ▸).

## 4. Software and data analysis

I16 users carry out a wide range of experiments, so a single, universal data-processing pipeline is not practical. Instead, data analysis is typically tailored by users to the specific needs of their experiments. To support this, the beamline and data-analysis teams have developed a suite of tools that help users analyse and visualize their data quickly and reliably. These tools are openly developed, with collaboration encouraged, and their source code available through the online repositories cited in the following sections. In most cases, they can also be used at users’ home institutions. Below are some examples of the tools available to users of I16.

### 4.1. Data manipulation–*MMG Toolbox*

The *Magnetic Materials Group (MMG) Toolbox* (Diamond Light Source, 2026*b* ▸) is a collaborative project across the Magnetic Materials Group beamlines at Diamond Light Source. The *MMG Toolbox* includes a graphical user interface for viewing experimental data on I16, built on a previous beamline *Data Viewer* (Porter, 2020 ▸) and using the standard *TkInter* framework. The viewer reads NeXus (Könnecke *et al.*, 2015 ▸) files and takes advantage of the format’s automatic plotting features to display the appropriate axes and detector images. The viewer supports a range of simple data-analysis tasks, including peak fitting, plotting multiple scans (as multi-line plots, 2D images, or 3D surfaces) and generating Python scripts and *Jupyter Notebooks* to provide flexibility in data manipulation.

### 4.2. Auto processors

It is also possible to run Python-based *Jupyter Notebooks* automatically at the end of scans that perform automated processing and analysis of specific scan types. This can include data-file conversion, detector re-mapping, peak fitting and the generation of calibration data. Completed scan files and the resulting processed notebooks are made available to users through the SynchWeb platform and ISPyB (ISPyB, 2020 ▸).

### 4.3. Reciprocal-space mapping

Data from area detectors such as the Pilatus3 100k and Quad Merlin are often summed or reduced to small regions, but this can discard a significant amount of useful information.

**Figure 4:**
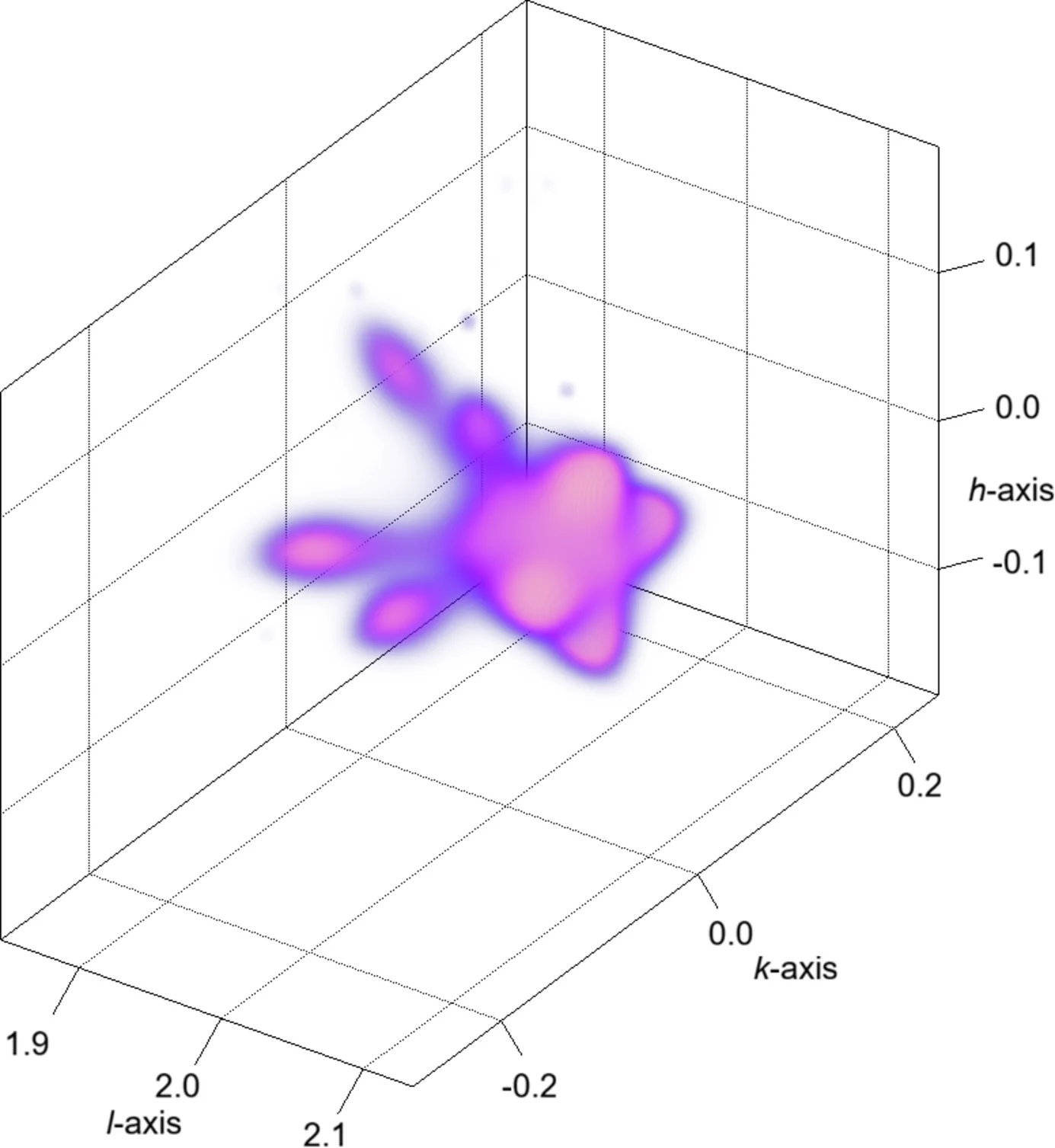
*MSMapper* processed reciprocal-space map of a Bragg reflection where peak splitting is observed.

Data collected on I16 are written in the NeXus file format (Könnecke *et al.*, 2015 ▸), adhering to the NXmx application definition, with some minor additions. This provides a complete description of the diffractometer and detector geometry using internationally agreed metadata standards. An important application of this approach is the remapping of detector images into a reciprocal-space volume. This enables users to visualize and analyse the shape of Bragg reflections relative to the sample’s basis vectors or surface. It also allows strain gradients along specific axes to be isolated and supports more sophisticated background-subtraction methods. Reciprocal-space volume generation can be carried out using *MSMapper* (Diamond Light Source, 2024*b* ▸) automatically—via *Jupyter Notebooks* triggered by adding a simple command to the scan—or performed afterwards using a user-friendly interface, resulting in outputs like that in Fig. 4 ▸.

### 4.4. MaRS

**Figure 5:**
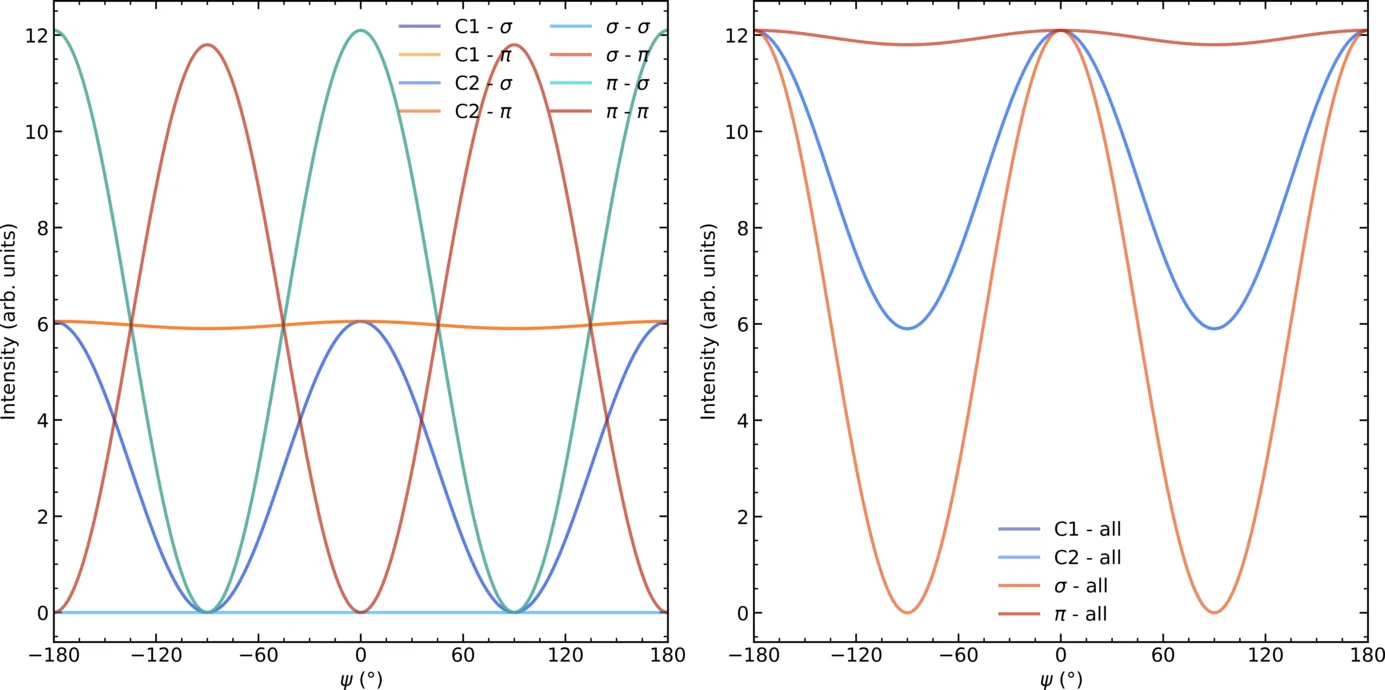
An example *MaRS* simulation output of the azimuthal dependence of a magnetic reflection (left) in a collinear structure with and (right) without final linear polarization analysis.

Beyond data manipulation, users at I16 have access to *MaRS* (Bombardi, 2020 ▸), a magnetic scattering simulation tool available as an API. This software package simulates the azimuthal dependence of REXS and NRXS for arbitrary magnetic structures within the dipole–dipole approximation and can be used to separate the spin and orbital contributions to the magnetic scattering. The results for the selected scattering process are presented as azimuthal dependence curves of the chosen reflection for each incident polarization, both with and without a final linear-polarization analysis as presented in the example in Fig. 5 ▸. Additional information can be found in Appendix *A*[App appa].

### 4.5. Reconstructing coherent diffraction data

The analysis of BCDI data follows a workflow that transforms raw 3D diffraction volumes into real-space images of nano-crystallite structure and lattice distortion.

This is supported on I16 by a suite of open-source software tools tailored for coherent-diffraction analysis using data from the beamline.

*Bonsu* (Newton *et al.*, 2012 ▸), an interactive phase-retrieval suite, provides a comprehensive environment for BCDI data reconstruction and visualization. It implements a wide range of iterative phase-retrieval algorithms and offers real-time visual feedback in both two and three dimensions, facilitating rapid optimization of reconstruction parameters.

An alternative workflow combines *CDIutils* (Atlan, 2020 ▸) and* PyNX* (ESRF, 2020 ▸; Favre-Nicolin *et al.*, 2020 ▸). *CDIutils* provides tools for pre-processing, detector geometry calibration, reciprocal-space mapping and parameter tuning for phase-retrieval pipelines. It acts as a high-level interface that prepares BCDI datasets for reconstruction. PyNX, a GPU-accelerated toolkit for coherent X-ray imaging, performs the computationally intensive phase-retrieval steps using operator-based algorithms optimized for both 2D and 3D CDI.

## 5. Science showcase

The I16 beamline offers a set of complementary scattering and coherent imaging techniques that are well suited to the study of a breadth of complex electronic, magnetic, and structural phenomena in material systems across different length scales. It is hence challenging to give an exhaustive overview of the full scientific scope of the beamline, and the following examples are intended to show some capabilities rather than define the limits of the instrument. In practice, the flexibility of the beamline, combined with ever developing experimental methodologies and the creativity of the user community, continues to expand the range of accessible experiments and scientific questions.

### 5.1. Ordering phenomena

Investigations of ordering phenomena are central to addressing many of the key problems in modern condensed matter physics. This is because establishing the ground state is a necessary step towards developing an understanding of the microscopic interactions governing the investigated system. The requirements for such studies are incredibly varied due to the breadth of the field which spans frustrated- and quantum magnets with complex magnetic textures and non-trivial topologies, multiferroics with multiple ordering parameters, charge order and charge density waves (CDWs), and orbital and molecular orders, amongst many others (Ramirez, 1994 ▸; Vasiliev *et al.*, 2018 ▸; Takagi *et al.*, 2019 ▸; Spaldin & Ramesh, 2019 ▸; Chen *et al.*, 2016 ▸).

**Figure 6:**
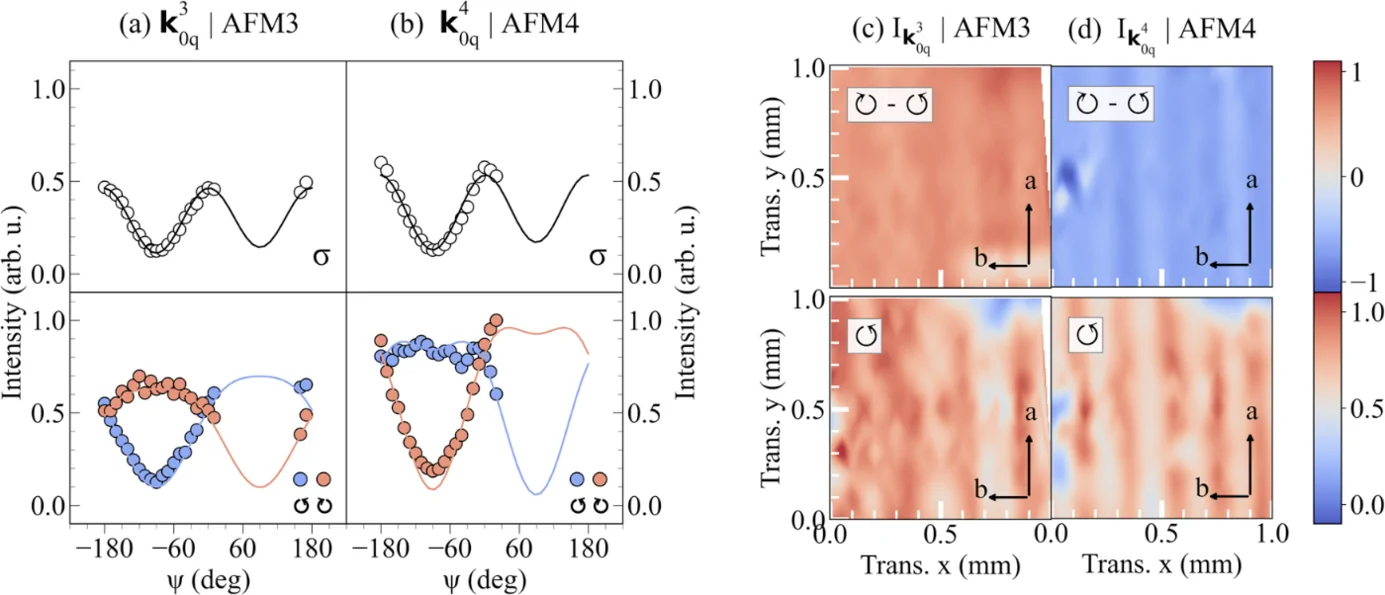
(*a*, *b*) Azimuthal dependence of the *k*^3^ = [0.165 (5),0,0] and *k*^4^ = [0.188 (5),0,0] satellites of the (0,0,8) reflection with linear horizontal (σ), circular left and circular right incident polarizations. (*c*, *d*) The variation in the difference in intensity of the satellite of the (0,0,8) reflection collected with circular left and right incident polarization as the beam was translated across the sample within the AFM3 and AFM4 magnetic phases, respectively. Figure adapted with permission from Vibhakar *et al.* (2024 ▸), copyright 2024 CC BY 4.0.

A first step in many investigations is the determination of the ordered state. The ability to combine resonant diffraction and polarization analysis allows for complex ordering patterns to be disentangled even in systems with multiple competing phases. A recent example is EuAl_4_, where REXS measurements established the complete sequence of four magnetic phases coexisting with charge density wave order (Vibhakar *et al.*, 2024 ▸). Furthermore, by translating the beam across the sample, the chirality of the helical magnetic structure was determined and a temperature-dependent reversal of the spin chirality between magnetic phases was observed (Fig. 6 ▸). Similarly, in 1*T*-TiSe_2_, resonant scattering measurements combining azimuthal scans with polarization and symmetry analysis on sets of space-group forbidden and non-forbidden reflections in the charge density wave state established the symmetry of the CDW and clarified the relationship between the structural and electronic order (Ueda *et al.*, 2021 ▸). The resulting picture resolved several contested features in the thermodynamic properties of the system and was found to be consistent with the absence of chirality in both the CDW and the orbital ordering pattern.

Beyond this, a further question concerns the extent to which ordered states can be manipulated through external stimuli. This can be examined on I16 using the available uniaxial strain cells. In CoTi_2_O_5_, for example, the application of uniaxial strain during a REXS experiment revealed near-complete switching between antiferromagnetic domains through the application of both compressive and tensile strain (Behr *et al.*, 2024 ▸). Combined with symmetry analysis, these measurements established the presence of the spin Jahn–Teller effect and the role of strain in biasing the system towards a single *k* domain.

Many of these studies require access to absorption edges in the tender X-ray regime. The ability of I16 to operate down to 2.46 keV while retaining its full suite of capabilities has enabled investigations at the U *M*_4,5_ and Ru *L*_2,3_ absorption edges. Examples include studies of magnetoelastic coupling in UN and U_2_N_3_ thin films (Lawrence Bright *et al.*, 2019 ▸), a review of the orbital and magnetic ordering mechanisms in Ca_2_RuO_4_ (Porter *et al.*, 2018 ▸), strain tuning of the magnetic structure in Ca_3_Ru_2_O_7_ (Dashwood *et al.*, 2023 ▸), and a recent investigation of RuO_2_ (Occhialini *et al.*, 2026 ▸).

Beyond determining the ordered states lies the challenge of identifying the microscopic interactions responsible for stabilizing them. An example is the investigation of the interference between non-resonant magnetic scattering and resonant quadrupole scattering, which allowed for the determination of the phase of the magnetic scattering amplitude and, consequently, the sign of the Dzyaloshinskii–Moriya interaction in a series of 3*d* transition metal [*M*CO_3_, *M* = Ni, Co, Mn, Fe] weak ferrimagnets (Beutier *et al.*, 2017 ▸).

**Figure 7:**
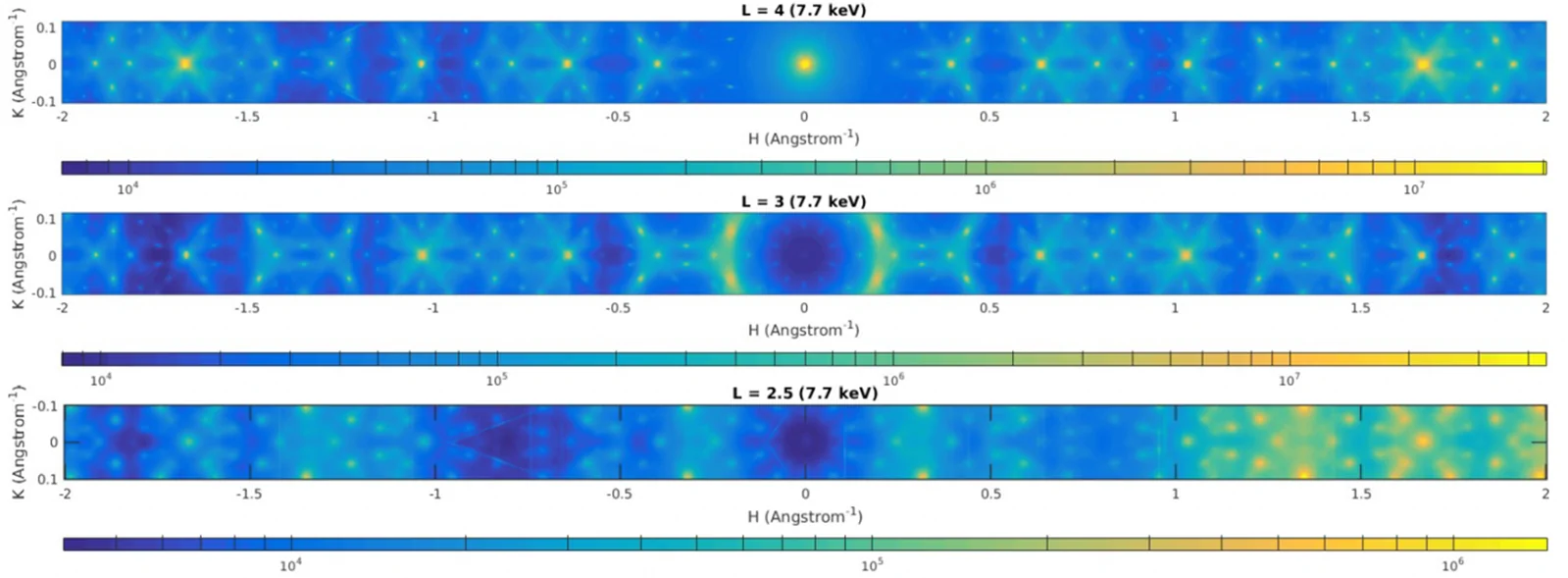
Reciprocal-space maps of the (top) *L* = 4, (middle), *L* = 3 and (bottom) *L* = 2.5 layers in the decagonal quasicrystal of Al–Co–Ni alloy highlighting the non-resonant diffuse signal measured at 7.7 keV (Beutier, 2026, private communication).

While much of the discussion thus far concerns long-range order, many important materials show strong local correlations. These can be probed through diffuse scattering measurements on I16 due to the combination of high flux with in-vacuum photon counting detectors. An example is an investigation of forbidden reflections arising from the anisotropy tensor of scattering (ATS) in a decagonal Al–Co–Ni quasicrystal (Beutier, 2026, private communication). In addition to the observation of the intensity arising from ATS, strong resonant and non-resonant elastic diffuse scattering is seen in Fig. 7 ▸.

While REXS is commonly used to investigate ordering phenomena, non-resonant X-ray scattering can offer advantages in particular cases. Specifically, one of the main advantages of non-resonant magnetic scattering is the possibility of separating the spin and orbital components of the magnetization. NRXS can also allow for an easier interpretation of the magnetic scattering cross section by avoiding the complexity of the resonant matrix elements. Given the weak nature of some investigated scattering processes, NRXS can be specifically useful as it adds an additional degree of freedom-the incident photon energy-that can be used to minimize contamination from, for example, multiple scattering. Additionally, NRXS can be chosen to minimize parasitic signals like multiple scattering or fluorescence by choosing optimal measuring conditions to maximize the signal to noise ratio. This was exactly the case for a study on 1 µm-thick BiFeO_3_ thin films (Waterfield Price *et al.*, 2016 ▸), for example, where substrate-induced strain and electric polarization were used as tuning parameters to manipulate magnetic domains.

#### 5.1.1. Soft condensed matter

A defining characteristic of many soft condensed matter systems is the presence of hierarchical order extending across multiple length scales, from molecular packing and nanoscale organization to mesoscopic and macroscopic structures. Understanding how these structural motifs interact and evolve is central to a wide range of problems involving polymers, liquid crystals, colloidal assemblies, membranes, nanocomposites and organic electronic materials (Aoki *et al.*, 2025 ▸), amongst many others. Experimental studies therefore require access to a broad range of scattering vectors while maintaining flexibility in sample geometry and environmental control.

I16 is particularly well suited for such soft matter studies as it supports specular and off-specular reflectivity, grazing-incidence scattering and diffraction (GISAXS and GIWAXS), transmission SAXS and WAXS measurements on both oriented and powder samples, and full single-crystal diffraction. Together with the energy tunability and rapid automated energy changes, and with detector distances ranging from 250 to 2000 mm, these capabilities allow for experiments to be optimized for SAXS, WAXS, or simultaneous SAXS and WAXS coverage. Depending on the chosen configuration, *Q* ranges from 0.007 nm^−1^ to 360 nm^−1^ can be accessed, corresponding to real-space length scales of approximately 900 nm to 0.017 nm.

Liquid crystals provide an excellent example of such investigations. Recent studies on I16 combining transmission and grazing-incidence SAXS/WAXS measurements have investigated twist-bend, ferroelectric nematic, bicontinuous cubic, and non-cubic phases that show spontaneous chirality (Li *et al.*, 2022 ▸). These experiments showed that the spontaneous formation of chirality through the twisting of molecular orientations, previously established in columnar segments of bicontinuous phases, also occurs in columnar phases. The resulting structures comprise complex three-dimensional arrangements of left- and right-handed columns whose organization is governed by the competing requirements of chirality and efficient space filling.

**Figure 8:**
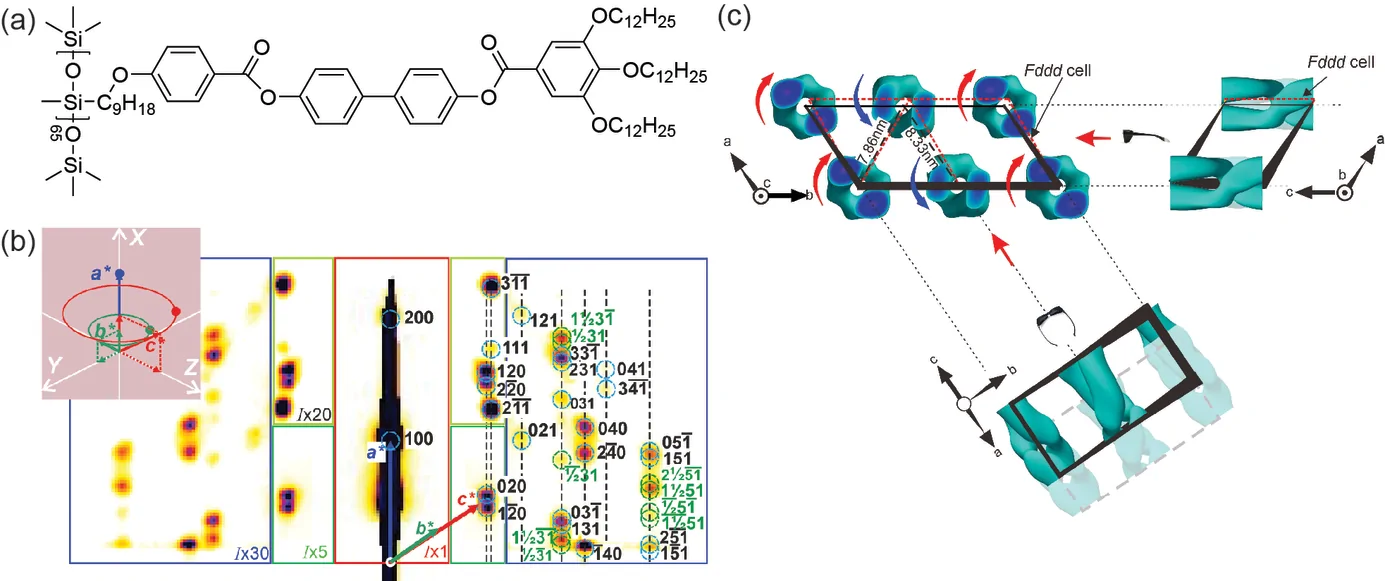
(*a*) Chemical structure of the side-chain LC polymer. (*b*) Grazing incidence X-ray diffraction pattern revealing the triclinic lattice. (*c*) Reconstructed electron density maps of the triclinic phase viewed in different directions.

More recently, other self-assembled structures have been identified in side-chain liquid-crystal polymers (Xue *et al.*, 2025 ▸). In these systems, left- and right-handed double-helical columns form highly ordered assemblies that can undergo additional distortions leading to triclinic symmetry (Fig. 8 ▸).

### 5.2. Imaging and dynamics at the nanoscale

While conventional diffraction is fundamental for determining the average structure of a material, many physical phenomena are governed by defects and fluctuations to the local environment that are not captured in such measurements. Instead, these are often explored using microscopic and spectroscopic techniques. BCDI techniques, where the transverse coherence length of the incident beam exceeds the dimensions of the sample, offer an alternative route through which a three-dimensional projection of the displacement field, and hence a spatially resolved phase-contrasted image of the sample, can be retrieved from the diffraction pattern (Robinson & Harder, 2009 ▸; Sun & Singer, 2024 ▸). These measurements can be done on both polycrystalline samples, where single grains are isolated for study, and to tailored samples such as focused ion beam fabricated single crystals.

**Figure 9:**
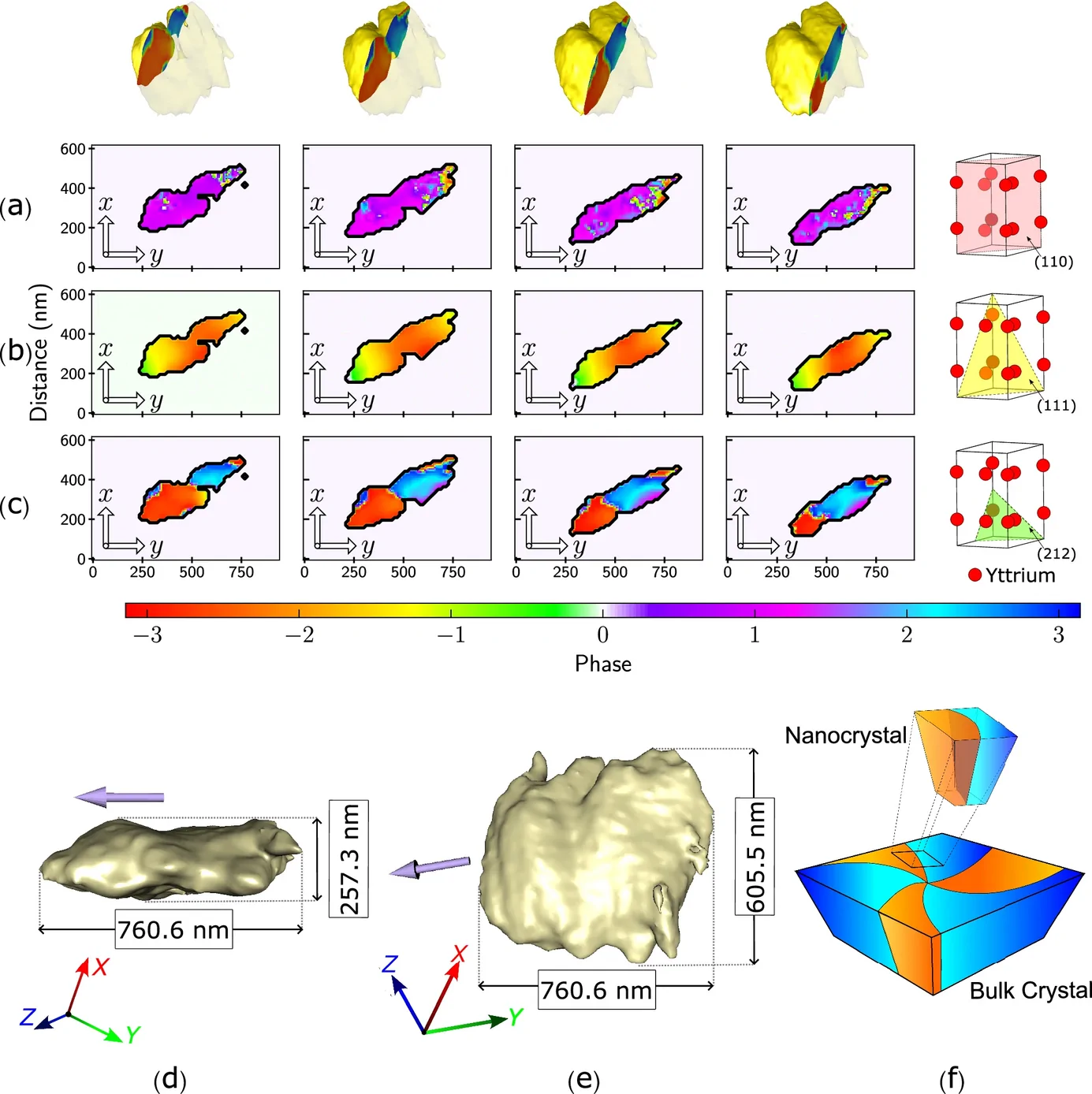
(*a*, *b*, *c*) Two-dimensional slices illustrating the reconstructed phase and amplitude of the (1,1,1), (1,1,0), and (2,1,2) reflections, respectively. (*d*, *e*) Three-dimensional reconstructions of the measured crystal. (*f*) Illustration of the ferroelectric domain formation in YMnO_3_. Figure adapted with permission from Mokhtar *et al.* (2024 ▸), copyright 2024 CC BY 4.0.

BCDI experiments yield a single projection of the strain within the sample, and can be further expanded to determine the full strain tensor through multi-reflection BCDI (Newton *et al.*, 2010 ▸). This makes BCDI experiments on I16 particularly suited for characterizing a broad set of phenomena spanning *in operando* battery cells (Serban *et al.*, 2024 ▸), structural transitions under external stimuli (Yang *et al.*, 2025 ▸; Newton *et al.*, 2016 ▸), and domain formation in ferroelectrics (Mokhtar *et al.*, 2024 ▸) and multiferroics (Najeeb *et al.*, 2025 ▸) (Fig. 9 ▸), amongst many others. This includes a dynamic BCDI study examining the crystal growth and dissolution cycles of calcite which highlights the effects of dislocations on the morphology of a crystal during its growth process (Clark *et al.*, 2015 ▸).

Coherent diffraction imaging techniques are not limited to studying the defect structure of materials, but can also be used to examine local structural dynamics at various timescales with momentum resolution through XPCS. This is done by correlating a series of coherent diffraction patterns across the time domain to obtain the intensity-intensity autocorrelation function (Shpyrko, 2014 ▸; Sandy *et al.*, 2018 ▸). On I16, XPCS has been used to examine a broad range of phenomena, including the structural dynamics across a second-order structural transition in the Heussler alloy AuAgZn_2_ (Livet *et al.*, 2015 ▸), the atomic relaxation dynamics across the CDW transition in a LSCO system (Shen *et al.*, 2023 ▸), and the dynamics of magnetic domain formation in USb (Lim *et al.*, 2014 ▸).

### 5.3. Beyond conventional diffraction

**Figure 10:**
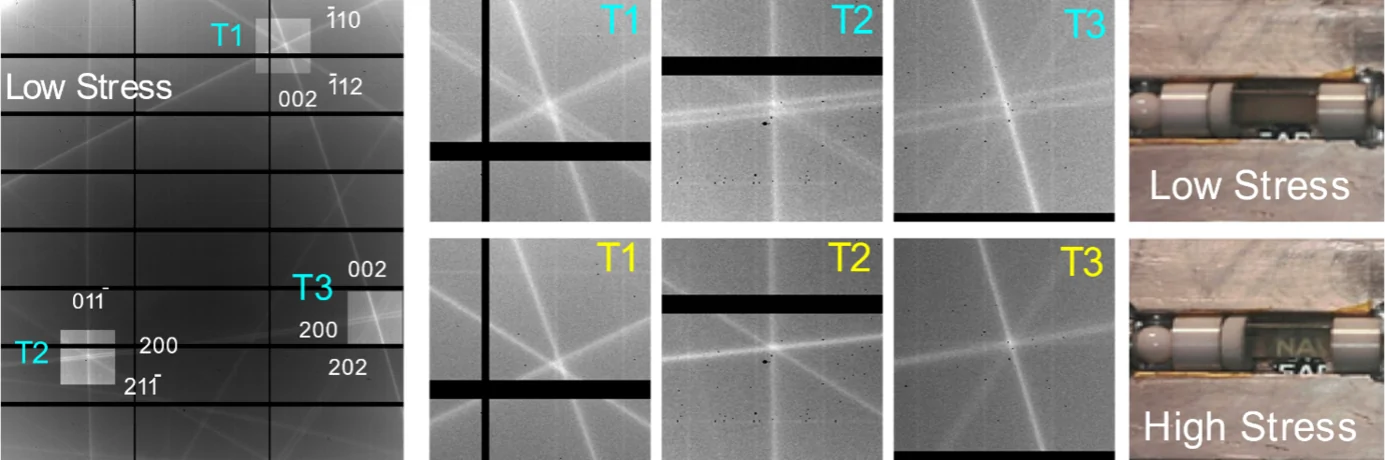
(Left) Pilatus 2M detector image at the (1.568, 1.214, 3.976) non-integer reflection measured at 9.63 keV with an applied mechanical stress of 6.8 MPa. Three regions are highlighted and enlarged for clarity. T1, T2 and T3 (cyan) represent the line intersections of coplanar reflections. T1, T2 and T3 (yellow) represent the same line intersections at high stress. (Right) Illustration of the sample being opaque and transparent at low and high stress, respectively. Figure adapted from Nisbet *et al.* (2023 ▸).

I16 has served not only as a user facility but also as a platform for the development and application of unconventional scattering methodologies. One particular example concerns the development of the theoretical and mathematical frameworks surrounding diffuse multiple scattering (DMS) (Nisbet *et al.*, 2015 ▸; Estradiote *et al.*, 2025 ▸). Here, one examines the diffuse scattering events that further undergo Bragg diffraction, resulting in well defined lines of scattering through which deviations from ideality in crystals can be monitored (Fig. 10 ▸). Such measurements provide a means to probe local structural disorder and correlations, and can, for example, be used to discriminate between phases in complex solid multi-phasic systems (Nisbet *et al.*, 2023 ▸). Additionally, DMS can be further extended to the structural characterization of functional materials under external stimuli. For instance, DMS has been used to track the phase evolution of PIN-PMN-PT across its ferroelectric transitions under combined stress and electric field cycling, confirming the high-stress phase to be monoclinic (Finkel *et al.*, 2022 ▸).

I16 has proved an ideal playground for investigating unusual effects such as in Borrmann spectroscopy where a huge relative enhancement of quadrupole absorption in thick-crystal Laue forward-diffraction is shown to be extremely sensitive to thermal displacements of the resonant ion (Collins *et al.*, 2009 ▸). Similarly, thermal motion induced resonant forbidden scattering (Beutier *et al.*, 2012 ▸; Beutier *et al.*, 2015 ▸) has shed light on the evolution of the electronic ground state with atomic displacement, leading to an unusual temperature dependence of the scattering signal that shows a sharp increase with temperature.

Beyond this, I16 has also been used to perform spectroscopic measurements such as the parametric down conversion of X-rays into pairs of lower energy, X-ray, visible or ultraviolet photons within crystals. Such experiments can provide simultaneous access to structural and spectroscopic information that probe the atomic scale charge density and electronic structure, making them particularly useful for photonics and for examining charge ordering phenomena (Sofer *et al.*, 2019 ▸; Sofer *et al.*, 2021 ▸).

## 6. A look into the future of I16

The upgrade of Diamond Light Source to the fourth-generation Diamond-II storage ring represents a significant step forward for I16. Operating at 3.5 GeV with a reduced horizontal emittance, the upgraded machine is expected to deliver an increase in spectral brightness of approximately one order of magnitude and an increase in coherent fraction approaching two orders of magnitude across the beamline operational energy range. Additionally, the beam delivered to the sample position is expected to become more symmetric, with dimensions approaching 50 µm (H) × 20 µm (V). These improvements are accompanied by an increase in the power density incident on the first crystal of the channel-cut monochromator by a factor of approximately 1.8, reaching around 44 W mm^−2^. The current monochromator design will not be able to accommodate this increase, and a new system is therefore being developed for installation prior to Diamond-II operation. The upgraded monochromator design will provide an opportunity to extend the accessible energy range, particularly into the tender X-ray regime. Together, these developments will enhance the performance of the existing techniques while opening new opportunities for coherent imaging and scattering experiments.

**Figure 11:**
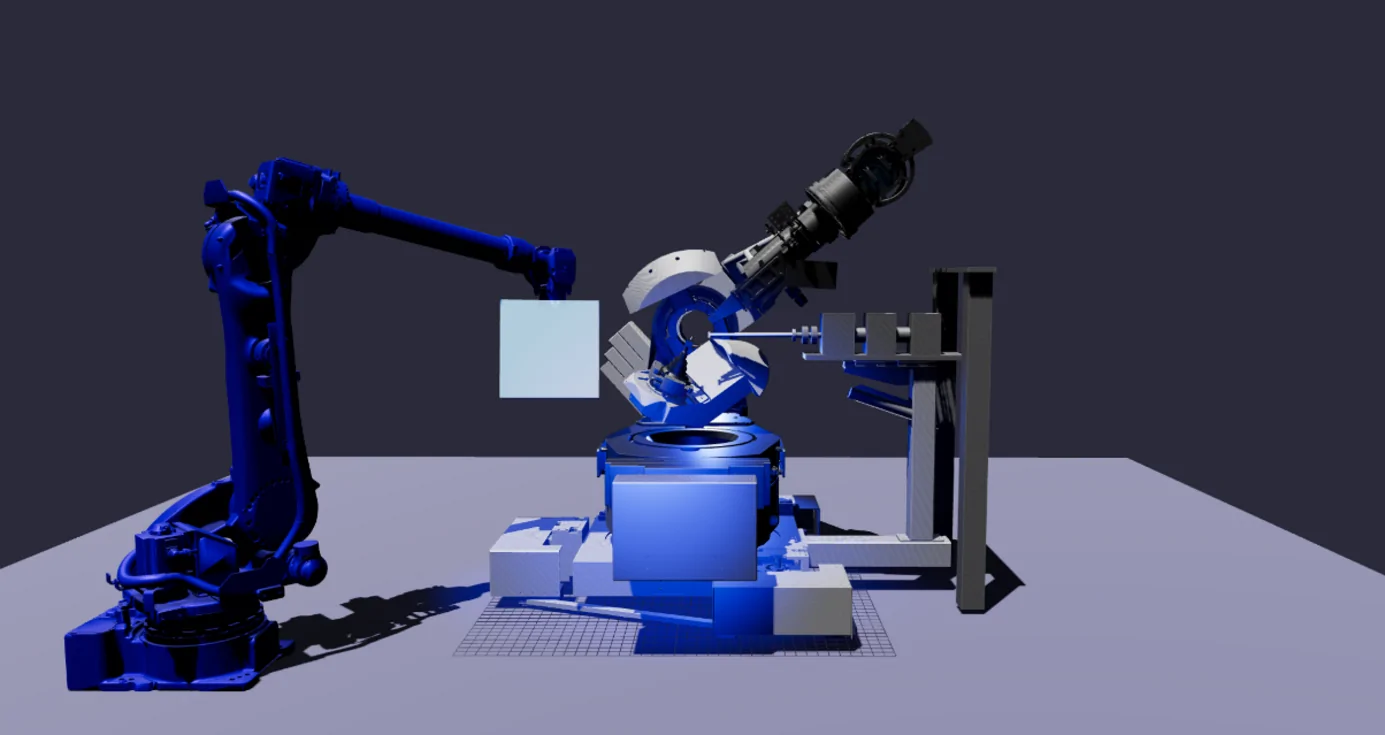
Simulation of the I16 diffractometer with the newly installed Yasakawa GP180-120 robotic arm.

Alongside the source upgrade, several developments are simultaneously happening on the beamline. A key development is the installation of a Yaskawa GP180-120 robotic detector arm. A simulation of the new configuration is presented in Fig. 11 ▸. Initially equipped with a Pilatus 2M detector, the new system will increase the efficiency and automation of data collection and expand the experimental opportunities across all research areas of the beamline. The large-area detector coverage will enable rapid sample screening, orientation determination, and reciprocal-space mapping with minimal beamline reconfiguration. Experiments that currently require multiple detector configurations, such as diffuse multiple scattering measurements, will become substantially more efficient. Similarly, transitions between diffraction, GISAXS, and GIWAXS measurement modes will be streamlined, increasing experimental throughput and allowing for more comprehensive investigations during a single beamtime allocation. Moreover the detector robotic arm will utilize the full length of the experimental hutch, thus extending the available *Q* range.

The extended reciprocal-space coverage will also enable the use of the Pilatus 2M for temperature-dependent measurements, allowing one to track any subtle lattice distortions associated with magnetic transitions more easily. Furthermore, this will also enable more quantitative studies of structural domain formation and ordering phenomena, such as charge density wave transitions, by collecting large numbers of reflections. Looking beyond the initial stage of the robotic arm, integrating a more modern detector with smaller pixel size will extend the possibilities of performing coherent imaging experiments with better resolution at varying energies. This will allow for BCDI on micrometre-sized crystals

To reduce data-collection dead time, the diffractometer control system is being upgraded to support continuous trajectory scans in both real and reciprocal space. By eliminating stepwise motion overheads, these developments will enable data of comparable quality to be collected in a fraction of the time.

As experimental complexity increases, the short and long term beam stability becomes increasingly important. To address this, automated procedures are being developed to continuously monitor the position and shape of the beam after each active optical element. Recording these positions will form a reference for reaching a fully automated alignment procedure with few different beam size choices matching the specific scientific case.

It is almost impossible to not include in the future of I16 the incorporation of AI agents to several aspects of the beamline life. Here we limit to mention that the procedure to search and distinguish amongst complex ordering phenomena will be made more efficient using AI driven symmetry analysis and on the fly analysis that will permit more exhaustive and real-time guided experiments.

## Figures and Tables

**Table 1:** List of axes operated in Eulerian geometry on the the six-circle Kappa diffractometer on I16

Angle	Description	Range (°)
η	Vertical geometry θ	−20 to 110
δ	Vertical geometry 2θ	−10 to 130
ϕ	Sample rotation	0 to 360†
χ	Angle ⊥ to incident beam	−100 to 100
μ	Horizontal geometry θ	0 to 100
γ	Horizontal geometry 2θ	0 to 110

† Range is restricted to < 200° with cryostat installed.

## Appendix A MaRS

The process of understanding the magnetic scattering cross-sections from resonant and non-resonant X-ray scattering measurements is often left as a time-consuming and difficult post-experiment task. Thanks to the recent development of a magnetic scattering simulation tool, *MaRS* (Bombardi, 2020 ▸), this can now be performed during or before beamtime, allowing users to better plan experiment strategies and understand their magnetic structures during the beam time. The *MaRS* software package is available as an API (Bombardi, 2020 ▸).

The software package simulates the geometric dependence of resonant elastic X-ray scattering for arbitrary magnetic structures within the dipole–dipole approximation. *MaRS* can also model non-resonant magnetic scattering as well as separating the spin and orbital contributions to the magnetic scattering.

Sample orientation is specified by the user in terms of the specular reflection and the azimuthal reference chosen for their experiment. The incident radiation is handled using the density-matrix formalism, and results are automatically produced for the standard polarizations used on the beamline: linear vertical, linear horizontal, circular left (C1), and circular right (C2). The beam is assumed to be fully polarized, and users provide the photon energy and select whether the calculation is resonant or non-resonant.

Magnetic structures and interaction vectors follow the conventions of the *FULLPROF* suite (Rodríguez-Carvajal, 1993 ▸; Rodriguez-Carvajal *et al.*, 2025 ▸) to ensure consistency with widely used tools in the magnetic scattering community. As in *FULLPROF*, the magnetic structure is expressed in terms of its decomposition in Fourier components and of a propagation vector. This gives users the flexibility to use symmetry-adapted modes, constraining the number of free parameters and allowing for a systematic exploration of candidate magnetic textures, or to retain a fully general description when needed.

Users can then provide the crystallographic information for the magnetically active ions in the crystal. Within this formalism, the user defines a basis consisting of an arbitrary number of complex vectors associated with one or more magnetic ions. Each complex vector contains seven parameters, which include the real and imaginary parts of the *S*_*k*_ components along the three orthogonal axes, plus a global phase. These vectors define how the magnetic moments transform within the chosen coordinate system.

Up to six Fourier components can be defined for each magnetic ion in the structure, allowing one to explore completely unconstrained solutions. In practice, however, the number of free parameters can often be reduced by applying physical or symmetry based considerations such as the size of the magnetic moment, a propagation vector that restrict Fourier components to be real and symmetry relations linking ions within the structure.

Users may also choose to express the magnetic structure in a crystallographic or spherical basis to simplify the description. In such cases, the necessary basis transformations are handled internally by the software. A built-in visualization tool is also provided to confirm that the real-space magnetic structure generated by the model is consistent with the reciprocal-space description supplied by the user.

Final results for the selected scattering process are presented as azimuthal dependences of the chosen reflection for each incident polarization, both with and without a final linear-polarization analysis (Fig. 5 ▸). In the web application, the analyser is assumed to be perfect, but the backend version allows users to specify the actual analyser angle to account for polarization cross-talk.

Several sanity checks are performed to verify the congruence between the various input parameters. For example, the number of basis vectors must match the number of supplied amplitudes, and the chosen reflection must be compatible with the propagation vector.

To keep the web interface simple, several advanced features of *MaRS* are available only through the backend Python package. This package is included in the *Jupyter Notebook* automatically generated at the start of each experiment on I16 and also provides tools for fitting azimuthal-scan data.
